# Effects of Transport at Weaning on the Behavior, Physiology and Performance of Pigs

**DOI:** 10.3390/ani4040657

**Published:** 2014-10-27

**Authors:** Mhairi A. Sutherland, Brittany L. Backus, John J. McGlone

**Affiliations:** 1 AgResearch Ltd., Ruakura Research Centre, Hamilton, 3214, New Zealand; 2 Animal Care Services, Texas Tech University, Lubbock, TX 79409, USA; E-Mails: brittany.backus@ttu.edu (B.L.B.); john.mcglone@ttu.edu (J.J.M.); 3 Department of Animal and Food Sciences, Texas Tech University, Lubbock, TX 79409, USA

**Keywords:** behavior, stress, pigs, transport, weaning, welfare

## Abstract

**Simple Summary:**

Pigs are commonly transported to separate production facilities at weaning to reduce disease transfer, enhance productivity and to improve overall operational efficiency. A review of the scientific literature suggests that these animals experience stress due to concurrent weaning and transport; however, gaps in the knowledge include the short and long term health and welfare implications of transporting pigs at weaning. Pig welfare and the efficiency of the swine industry may improve if science-based recommendations were in place.

**Abstract:**

Transport of pigs to separate production facilities at the time of weaning is a common practice, primarily performed to reduce vertical transfer of disease and enhance production and overall farm efficiency. During transport, pigs are exposed to numerous stressors in conjunction with the stress experienced as a result of weaning. In this review, the behavioral and physiological response of pigs experiencing weaning and transport simultaneously will be described, including the effects of space allowance, season and transport duration. Based on the scientific literature, the gaps in the knowledge regarding potential welfare issues are discussed. Changes in behavior and physiology suggest that weaned pigs may experience stress due to transport. Space allowance, season and duration are aspects of transport that can have a marked impact on these responses. To date, the literature regarding the effects of transport on weaned pigs has primarily focused on the short term stress response and little is known about the effects of concurrent weaning and transport on other aspects of pig welfare including morbidity and mortality rates. Greater understanding of the short and long term consequences of transport on weaned pig welfare particularly in relation to factors such as trip duration, provision of feed and water, and best handling practices would benefit the swine industry. Furthermore, the development of guidelines and recommendations to enhance the short and long term welfare of weaned pigs in relation to transport are needed.

## 1. Introduction

Transport of pigs at weaning is a common practice in the USA and other countries. Pigs are predominantly transported to separate production facilities at weaning to reduce vertical transfer of disease and to improve early post-weaning growth and productivity potential of pigs [1]. In the USA, weaned pigs are typically 4–6 kg and 17 to 28 days of age. In other regions of the world, piglets may be older when weaned and transported. In the USA and Canada, weaned pigs are typically transported throughout the year in trailers without supplemental heat, feed or water and for durations that can range from 1 h to 24 h [2,3]. Besides breaking disease transmission between the sow buildings and the weanling buildings, transport to a unique nursery/finishing site allows for specialized care of the weaned pigs which enhances their health and welfare. An alternative system where weaned pigs remain in a farrowing environment (avoiding the need to transport piglets) may work well for smaller farms, but such systems are not common on modern, larger-scaled pig production units around the world.

During transport pigs are potentially exposed to numerous stressors including handling at loading and unloading, fluctuating temperatures, mixing with unfamiliar pigs (and ensuing social stress), feed and water withdrawal, exposure to a novel environment, vibrations and noise [4], which can lead to reduced welfare and increased morbidity and mortality. In market weight pigs, transport can cause stress as indicated by elevated cortisol levels, an increase in the lymphocyte to neutrophil (NL) ratio and heart rate [5,6], muscle exertion or injury as indicated by increased creatine kinase (CK) and lactate dehydrogenase concentrations [5,7] and morbidity and mortality [8,9].

Pigs transported at weaning are likely to experience similar stressors as older pigs, in addition to the stress associated with weaning. Weaning is a substantial stressor caused by the loss of the maternal experience (milk, pheromones, touching/comforting), and abrupt social, nutritional and environmental changes [10]. Weaning can affect the behavior, physiology and performance of pigs [1,11,12,13]. Therefore, it would be beneficial for pig welfare and the swine industry to understand the impact of transport on the health and welfare of weaned pigs, and where potential welfare comprises may occur so that it can be managed appropriately.

The objective of this review is to describe the general behavioral and physiological response of weaned pigs to transport. Known aspects of transport that have the potential to negatively impact the welfare of weaned pigs, including space allowance, season and transport duration will be discussed and the response of weaned pigs to these aspects of transport will be described. Based on the available scientific literature, the gaps in our knowledge regarding potential welfare issues associated with transporting weaned pigs will be discussed, and areas where more research is needed will be highlighted.

## 2. General Effects of Weaning and Transport

Weaning is a necessary part of the biology of mammals, although commercial production systems wean abruptly at an early age (≈17 to 28 days of age) relative to the gradual weaning observed in domestic sows in free-ranging conditions which occurs at approximately 17 weeks of age [14]. At weaning piglets are separated from their mothers and are exposed to dramatic environmental and social changes, such as change in diet and mixing with unfamiliar pigs, which can result in a range of behavioral, physiological and performance changes [1,11,12,13].

Behavioral changes associated with weaning include increased belly-nosing, resting and vocalizations and reduced feeding [12]. In addition, pigs are commonly mixed with unfamiliar pigs at weaning, which results in short but intense bouts of fighting until the dominance order of the group is established [15]. Age at weaning can influence the behavioral response of pigs; pigs weaned at 7 days of age spent more time belly-nosing and performing escape attempts and less time feeding than pigs weaned at 14 or 28 days of age [16]. An increase in behaviors such as vocalizations and escape attempts may signify a stress response in pigs [16], whereas reduced feeding is likely related to delayed adaption to a novel feed source.

Weaning in pigs is associated with physiological changes such as activation of the hypothalamic-pituitary-adrenal (HPA) axis [17], shifts in the sympathetic nervous system [11] and alterations in growth-related hormone profiles [18]. Weaning also causes changes in intestinal morphology, such as a reduction in villus height, increased villus width and increased crypt depth, and the enteric immune system which can ultimately decrease nutrient digestion and absorption, contribute to post-weaning diarrhea and increase susceptibility to enteric infections [1,19,20]. As weaning is a combination of stressful events it is difficult to separate out the effects of these different stressors. However, mixing of unfamiliar pigs alone can cause changes in physiological indices of stress such as elevated heart rate, increased secretion of cortisol and catecholamines, increased NL and reduced growth rates [21], whereas changes in IGF and gut morphology are more likely to be associated with the initial reduction in feed intake post-weaning and change in diet.

Reduced weight gain is commonly observed in pigs post-weaning and this response is greater in earlier weaned pigs. Pigs weaned at 1 week of age were found to take 12 h to 48 h to start consuming dry food [22] and had markedly reduced growth rates than pigs weaned at 14 or 28 days of age [16]. Reduced weight gain in pigs post-weaning is likely due to a combination of factors including reduced feed intake and lower feed quality/quantity as compared to what the pigs were receiving from their mother’s milk [12]. At the time of weaning, especially in the case of early weaning, the immune and digestive systems of pigs are not fully mature, which can make them more susceptible to nutritional or microbial challenges especially in combination with reduced feed intake [1]. Hence, early-weaned piglets are commonly transported to separate production facilities to reduce pathogen challenges [1].

Studying the welfare of weaned pigs in response to transport is complicated by the fact that they are also experiencing weaning, which is a significant and unique stress in itself. Any response of weaned pigs to transport must include consideration of the weaning experience. For example, pigs (and other livestock) are typically not provided feed or water during transport (unless the trip is very long, e.g., over 28 h in the USA, or over 8 h in the EU). Therefore, it is likely that the absence of feed and water while transporting weaned pig is of little consequence if the transport duration is less than 12 h because the weanlings would not normally be eating or drinking at this time even if they were not transported. However, these pigs are likely to still experience other aspects of weaning stress during transport including exposure to novel environments, and separation from the sow and familiar conspecifics.

Exposure to multiple concurrent stressors in growing pigs can have an additive detrimental effect on performance, hematological measures and behavior [23,24]. Because weaning is a unique and powerful stressor, any additive or interactive effects must be considered. Generally, the USA swine industry believes that weaned pigs do not experience the same level of stress as that of slaughter weight or adult pigs during transport because mortality is low during transport of weaned pigs and because the effects of weaning alone are so great while any added stressor would be less severe. However, research is needed to further investigate the welfare implications of transporting pigs at weaning using a multi-disciplinary approach that assesses pig behavior, physiology and performance, including morbidity and mortality rates.

## 3. Effect of Concurrent Weaning and Transport on the Behavior, Physiology and Performance of Pigs

Behavior of weaned pigs during [2,3,25,26] and in the days following transport [2,27] has been investigated (Table 1). The behavioral responses of weaned pigs during transport are relatively simple, they typically stand initially then lie down in the transport vehicle as transport duration increases [2,3]. Furthermore, weaned pigs spend more time resting in the days following transport compared to non-transported weaned pigs [2,27]. High levels of lying / resting during and after transport, especially after an initial period of standing, may indicate that pigs have become habituated to the novel conditions and/or are fatigued as a result of loading and transport [2]. Following transport, weaned pigs are slow to begin eating and drinking [2], perhaps due to exposure to a novel environment and/or food source (depending on whether they were creep fed in farrowing) and the lingering effect of the weaning experience. However, the time weaned pigs spent feeding and drinking increased with transport duration, suggesting that transport and the associated water and feed deprivation increased pigs motivation to eat and drink [2,27]. Space allowance, season and transport duration can affect the behavioral response of weaned pigs during and after transport [2,3,26], and increased resting, drinking and feeding behaviors after transport indicate that weaned pigs may experience dehydration, hunger and fatigue in response to transport [2,27].

Common physiological measures of stress in pigs include elevated cortisol concentrations, an indicator of activation of the HPA axis, and the NL. Both cortisol concentrations and the NL were elevated in weaned pigs after transport [3,25,26] indicating that concurrent weaning and transport causes acute stress in pigs.

**Table 1:** Summary of studies investigating the effect of transport on pigs at weaning.

Transport vehicle	Country	Age of pigs (d)	Space allowance (m^2^/pig)	Season	Duration (h)	Temperature (°C)	Feed and water available	References
Panel van	Canada	17 ± 1	Not stated	Summer	0, 6, 12 and 24	22.1 to 30.3	No	[30]
				Winter		−2.8 to 3.2		
Panel van	Canada	17 ± 1	0.06	Summer	0, 6, 12 and 24	22.1 to 30.3	No	[2]
				Winter		−2.8 to 3.2		
				Fall		3.4 to 12.8		
Van	Canada	17 ± 1	0.085 to 0.25	Summer	0, 6, 12 and 24	≈15 to 26	No	[29]
				Winter		≈−17 to 14		
Simulated	Canada	17 ± 1	0.094 to 0.28	Summer	0, 6, 12 and 24	≈15 to 26	No	[29]
				Winter		≈−17 to 14		
Van	Canada	17 ± 1	0.085 to 0.25	Summer	0, 6, 12 and 24	≈17.4 to 25.7	No	[27]
				Winter		≈−15 to 8.4		
Simulated	Canada	17 ± 1	0.094 to 0.28	Summer	0, 6, 12 and 24	≈19 to 23	No	[27]
				Winter		≈2.5 to 8.4		
Semi-trailer	USA	18 ± 1	0.05, 0.06 and 0.07	Summer	1	26.1 to 30.3	No	[3]
Semi-trailer	USA	18 ± 1	0.05, 0.06 and 0.07	Winter	1.9	−2.4 to 21.7	No	[25]
Semi-trailer	USA	18 ± 1	0.05, 0.06 and 0.07	Spring/Fall	2.5	14.9 to 29.5	No	[26]

Indicators of stress and/or tissue damage in slaughter weight and breeding age pigs in response to transport can include, elevated creatine kinase (CK) and aspartate aminotransferase (AST) concentrations [5,7,28]. Furthermore, blood urea nitrogen (BUN) is a waste product in the blood caused from the breakdown of protein. Sutherland *et al*. [3,26,27] found increased concentrations of CK, AST and BUN in weaned pigs immediately after transport suggesting that transport stress may cause a catabolic state due to the exertion of loading, unloading and transport itself.

Total plasma protein and albumin concentrations were greater in weaned pigs after transport [3,25,26], furthermore elevated hematocrit levels were found in weaned pigs transported in summer [3]. Total plasma protein and albumin are markers of protein homeostasis, which can increase with dehydration. Elevated total plasma protein and albumin concentrations and hematocrit levels suggest that weaned pigs may experience dehydration as a result of transport and the associated feed and water deprivation. In addition, body weight loss was greater after transport and weight loss increased with increasing transit time [29], further suggesting that weaned pigs can become progressively dehydrated as a result of longer transport and this could be exacerbated with elevated ambient temperatures.

Pigs lost an average of approximately 0.4 kg and 6% to 7% of their body weight in response to weaning with the minimum weight reached approximately 2 days post-weaning [29,30]. Transport, in particular transport duration negatively impacted weight loss of weaned pigs, but transport did not affect average daily gain or feed conversion efficiency for the 14 days post-weaning [29,30]. Weaning alone is known to result in increased morbidity and mortality in pigs [19,31] and morbidity and mortality rates associated with transporting slaughter weight pigs has been reported [8,9]. However, little is known about morbidity and mortality rates of weaned pigs in response to transport.

Changes in behavior, physiology and performance suggest that weaned pigs may experience acute stress, dehydration and protein break down due to transport. However, particular aspects of transport, including space allowance, season and transport duration, have the potential to exacerbate these responses.

## 4. Effect of Space Allowance during Transport

In the USA the recommended space allowance for transporting pigs up to 5.4 kg is 0.06 m^2^/pig [32], the Canadian Agrifood Research Council recommends 0.085 m^2^/pig for pigs up to 9 kg [29] and the European Food Safety Authority recommends 0.12 m^2^/pig for pigs up to 10 kg [33]. Space during transport should be evaluated differently than during a growing experience in a barn. If the floor space is too much, then pigs may fall and roll around in the truck or trailer, which may cause injury.

Space allowance during transport can directly affect the welfare of pigs. Giving slaughter weight pigs insufficient space during transport can be deadly or in the least cause serious injuries [34,35]. However, if the space is too large, pigs may have difficulty maintaining their balance when the truck stops, starts or makes turns [36] and potentially increase the risk of injuries during transit. Until recently, the space needs of weaned pigs during transport were not known.

The majority of scientific literature investigating the effects of space allowance on pigs during transport is focused on slaughter weight pigs [34,35,36]. Space allowance does not appear to markedly affect the physiology of weaned pigs during transport. Cortisol, AST, CK, and glucose concentrations, immune measures (e.g., neutrophil chemotaxis and phagocytosis) and performance measures (body weight gain and lesion scores) were not affected by space allowance [3,25,26] in weaned pigs. However, immediately after a 60 min transport period the NL was greater in pigs transported at a space allowance of 0.05 m^2^/pig compared with pigs transported at 0.06 or 0.07 m^2^/pig [3]. An increase in the NL is an indication of acute stress [37], hence pigs transported at a smaller space allowance may experience more stress than pigs provided with more space. The effect of space allowance on the NL was only evident in pigs transported in summer (26 °C to 30 °C) and not during mild or cold temperatures [25,26].

Furthermore, space allowance during transport can affect behavioral measures of stress and fatigue in slaughter weight pigs [34,36,38]. Weaned pigs transported at a space allowance of 0.07 m^2^/pig spent more time lying than pigs transported at 0.05 m^2^/pig or 0.06 m^2^/pig during a 60 min transport period in summer [3], suggesting that pigs transported at higher stocking densities are more restless possibly due to the lack of available lying space. Kim *et al*. [38] found that market weight pigs spent more time lying when transported at low (0.39 m^2^/100 kg) compared with medium (0.35 m^2^/100 kg) or high (0.31 m^2^/100 kg) stocking rates. On the contrary, weaned pigs transported at 0.06 m^2^/pig, in the spring and fall, spent more time lying than weaned pigs transported at 0.07 m^2^/pig [26]. However, space allowance did not affect lying times in pigs transported in winter [25]. Barton-Gade and Christensen [36] found that providing slaughter weight pigs with more space (0.42 m^2^/100 kg to 0.5 m^2^/100 kg) reduced lying times as a result of continuous disturbances from other pigs and pigs appeared to have more difficulty maintaining balance.

Other behaviors observed in weaned pigs during transport include sitting and standing/rearing, where pigs assume or maintain an upright position on extended legs while standing on another pig. Weaned pigs transported at 0.06 m^2^/pig in the spring and fall spent less time standing/rearing than pigs transported at 0.05 m^2^/pig or 0.07 m^2^/pig during a 148 min transport period [25]. Furthermore, weaned pigs transported at 0.05 m^2^/pig spent more time standing/rearing than pigs transported at 0.06 m^2^/pig or 0.07 m^2^/pig during a 60 min transport period in summer [3], and Sutherland *et al*. [25] found that weaned pigs spent less time standing/rearing when transported at a stocking rate of 0.07 m^2^/pig compared with 0.06 m^2^/pig during a 112 min transport period in winter. Weaned pigs also spent less time sitting when transported at 0.07 m^2^/pig compared with 0.05 m^2^/pig or 0.06 m^2^/pig during a 60 min transport period in summer [3]. Increased standing/rearing or sitting behavior may be an indication of competition for space or instability due to too much space [26]. Transported weaned pigs may benefit from slightly less space in cold weather and just a bit more space in warm weather. As a practical matter, transport trucks and trailers are a fixed size on a given farm and the numbers of pigs to be transported at a given time is also fixed. Adjusting the trailer floor space to more closely reflect the needs of the weaned pigs would benefit the weanlings.

Space allowance had more of an impact on weaned pig behavior during transport than physiological indices of stress. Ultimately, when weaned pigs were provided with more space they spent more time lying and less time standing. Furthermore, higher stocking densities resulted in pigs spending more time standing on other pigs. From these studies, it appears as though it is preferable to transport weaned pigs at a larger space allowance (e.g., 0.06 m^2^/pig to 0.07 m^2^/pig), but more research is needed to investigate the relationship between temperature/season and pig health and welfare at different space allowances during transport. Also, it may be beneficial to study the behavior of pigs during transport in more detail to distinguish between lying, lying/huddling (which may signify pigs are cold), or standing and standing/rearing. In future studies other measures of pig welfare, such as morbidity, skin lesions, body temperature and mortality rates may also be useful when developing recommendations for optimum space allowance for transporting weaned pigs.

## 5. Effect of Temperature/Season during Transport

Weaned pigs are transported year round over a range of ambient temperatures often in trailers that do not provide supplemental heat. Even though transport vehicles provide pigs some protection from ambient conditions in regards to shelter from wind and rain and bedding materials in the form of straw or wood shavings, temperatures within the trailers are still influenced by the outside environment. The effect of season on the behavior and physiology of transported weaned pigs has been investigated by Sutherland *et al*. [3,25,26], however in these studies it is difficult to separate the effects of season from transport duration. Lewis *et al*. [30] transported weaned pigs for 0 h, 6 h, 12 h or 24 h during three seasons, summer (22.1 °C to 30.3 °C), fall (3.4 °C to 12.8 °C) or winter (−2.8 °C to 3.2 °C). Season did not affect skin temperature, or weight loss or average daily gain (ADG) calculated from weights recorded daily during the first 7 days and on day 14 post-weaning [30]. In a similar study, Wamnes *et al*. [29] found that weaned pigs transported in winter had greater weight loss and lower ADG than pigs transported in summer, calculated from weights recorded daily during the first 8 days and on day 10, 12 and 14 post-weaning. Lewis *et al*. [30] found that pigs had lower core temperatures when transported in winter in Canada, suggesting that these pigs were unable to compensate for the cold temperatures, especially as no feed was provided during transport. Young pigs have poor thermoregulatory capacity due to a lack of brown adipose tissue and hence they rely predominantly on shivering to thermoregulate [39]. Therefore, increased weight loss could have resulted from pigs metabolizing more energy in order to maintain homeostasis. Providing less space during the winter or boarding the trailer (closing vents) may have increased the air temperature of the transport vehicle, whilst maintaining adequate ventilation. Because the winter-summer difference is likely to be an air temperature effect, providing a more comfortable (warmer) transport experience may have some benefit in the winter.

Lewis and Berry [2] investigated the behavior of weaned pigs during transport and in the 3 days following unloading over three seasons, summer, fall and winter. Weaned pigs spent more time resting and less time standing when transported in summer compared with fall and winter and this pattern was consistent over 24 h [2]. However in the 3 days following unloading, weaned pigs transported in the fall spent less time resting, standing and more time sitting in the home pen compared with pigs transported in summer or winter. In a similar study, Wamnes *et al*. [27] found that during the 4 days after transport, weaned pigs transported in summer spent more time lying than pigs that were transported in winter. Furthermore, weaned pigs transported in summer performed longer drinking bouts than pigs transported in winter in the home pen [27]. It is likely that pigs transported in summer experienced greater levels of dehydration as a result of elevated ambient temperatures and were therefore more motivated to drink following transport [27]. However, the motivation behind the seasonal variation in lying, standing and sitting are unclear.

## 6. Effect of Transport Duration

Transport duration has the potential to influence the well-being of pigs in transit. Interestingly in slaughter weight pigs, higher mortality rates were recorded during short (30 min to 90 min) transport times than transport times greater than 3 h [40]. Additionally, Sutherland *et al*. [41] found that the percentage of market weight pigs dead on arrival at the packing plant was greater when transport duration increased from 30 min to 4 h, and tended to decrease as travel durations extended longer than 4 h. Several studies have investigated the effect of transport duration on the physiology and behavior of weaned pigs. Lewis *et al*. [30] transported weaned pigs for 0 h, 6 h, 12 h or 24 h during three seasons, summer (22.1 °C to 30.3 °C), fall (3.4 °C to 12.8 °C) or winter (−2.8 °C to 3.2 °C) and found no effect of transport duration on weight loss or ADG (calculated from weights recorded daily during the first 7 days and on day 14 post-weaning) or skin temperature in pigs. However, Wamnes *et al*. [29] found an effect of transport duration on weight loss in weaned pigs with weight loss increasing with increasing transit time (performance measures were calculated from weights recorded daily during the first 8 days and on day 10, 12 and 14 post-weaning). Bryer *et al*. [42] found transport up to 30 h caused weight loss in replacement age gilts, however, they found a tendency for gilts transported at standard TQA recommended space allowances to lose more weight compared to gilts given 20% extra space at 18 h after transport, but not at 24 h and 30 h. Weight loss during transport is likely due to a combination of factors including loss of body water and gut contents, making pigs enter a catabolic state in order to maintain homeostasis.

The behavioral response of weaned pigs to transport changes over time; Lewis and Berry [2] found that weaned pigs spent more time resting during transit times between 12 h and 24 h compared to the first 12 h of travel and pigs spent approximately 76% of the time resting. However, Sutherland *et al*. [3] found that pigs spent approximately 68% of the time standing and 19% of the time lying when transported at a similar space allowance. During the first 45 min of transport, 88% of pigs were standing compared to 49% during the last 15 min of the trip [3]. It appears as though pigs become less restless, spend more time lying, as transport time increases, whether because pigs become habituated to transport or fatigued. The ability to determine whether pigs are lying more because of fatigue or because they have become habituated to the effects of transport would be useful when making recommendations in terms of optimum transport period for weaned pigs.

The effect of transport and transport duration can also influence the behavior of weaned pigs in the home pen after transport. During the first 3 days following transport, weaned pigs transported for 12 h or 24 h spent more time resting, feeding and drinking than control pigs [2]. Wamnes *et al*. [27] also found that weaned pigs transported for 12 h or 24 h spent more time resting and drinking than control pigs. Increased resting and drinking behavior performed by pigs transported for 12 h or more suggests increased motivation of these pigs to perform these behaviors, likely due to dehydration as result of extended water withdrawal, fatigue and weaning.

## 7. Gaps in the Knowledge and Conclusion

The effect of transport on the health and welfare of pigs has predominantly been studied in slaughter weight pigs. In comparison, few studies have investigated the effect of transport on weaned pigs. Primarily, the research has focused on the short term consequences of transport on weaned pigs, such as the immediate physiological stress response to transport, the behavior of pigs during and after transport and the performance of pigs up to 14 days post-transport. However, limited research is available on the morbidity and mortality of weaned pigs transported under different conditions, for example the effects of season or transport duration on pig mortality (e.g., number of pigs dead on arrival) or incidence of disease.

From information in the scientific literature, we can make some recommendations about best management practices for weaned pigs. For example, it appears to be preferable to transport weaned pigs at 0.06 m^2^/pig or 0.07 m^2^/pig rather than 0.05m^2^/pig, particularly in summer [3,25,26]. Furthermore, transporting weaned pigs for 12 h to 24 h without feed or water may result in pigs becoming dehydrated and/or fatigued [29]. However, the current research investigating the effects of transport on weaned pigs has focused on the short term behavioral and physiological responses of pigs. In future studies it would be beneficial to include other measures of pig welfare, such as morbidity, skin lesions and mortality rates. Aspects of handling and loading/unloading pigs, such as handling apparatus, group size and slope of ramps have been investigated in growing pigs [43,44,45], and based on the scientific literature recommendations and guidelines can be developed [46]. However, currently there are limited recommendations for handling or transporting weaned pigs. Research is needed on best practice for handling and moving, loading and unloading weaned pigs, for example optimum number of weaned pigs to move at one time, different apparatus to move pigs (e.g., sorting boards, flags, rattle boards) and slope of ramps when loading pigs into the trailer to reduce the number of slips and falls. Limited research on the need for boarding and bedding has been conducted in relation to transport during different seasons in slaughter weight pigs [47,48], but this information is completely lacking for transport of pigs at weaning.

In conclusion, changes in behavior, physiology and performance suggest that weaned pigs may experience stress due to transport, and space allowance, season and transport duration are aspects of transport that can have a marked impact on these responses. Transporting weaned pigs for up to 6 h may not be any more stress than just weaning alone, but after that, then they will become more dehydrated or lose more weight than weaning alone. It would be beneficial for pig welfare and the swine industry to understand more about the long term implications of transport on pig welfare, such as incidence of disease, and to develop recommendations to enhance the short and long term welfare of weaned pigs in relation to transport.
